# Incidence of Communicating Hydrocephalus Following Intraventricular Hemorrhage Among Adult Patients Treated at a Hospital in Jeddah, Saudi Arabia: A Retrospective Study

**DOI:** 10.7759/cureus.77699

**Published:** 2025-01-20

**Authors:** Abdulaziz A Alzahrani, Abdulrahman M Zawawi, Suhail H Alrudaini, Nader A Hassan, Adel A Alsulami, Abdulaziz M Alkhoshi, Mohammed Alyousef

**Affiliations:** 1 College of Medicine, King Abdulaziz University Faculty of Medicine, Jeddah, SAU; 2 Department of Neurosurgery, King Abdulaziz University Hospital, Jeddah, SAU

**Keywords:** glasgow coma score, hydrocephalus, intraventricular hemorrhage, length of hospitalization, ventriculoperitoneal shunt

## Abstract

Introduction

Intraventricular hemorrhage is a severe condition caused by bleeding within the brain ventricles. It is often due to trauma, tumors, vascular malformation, aneurysm, oxygen deprivation, or idiopathic. A common complication associated with intraventricular hemorrhage is hydrocephalus, which is the accumulation of cerebrospinal fluid in the ventricles. Hydrocephalus can be classified as communicating or non-communicating. This study aimed to evaluate the incidence of communicating hydrocephalus after intraventricular hemorrhage.

Methods

This retrospective study was conducted at King Abdulaziz University Hospital in Jeddah, Saudi Arabia, and included 52 adult patients treated between 2012-2022 who met the eligibility criteria. We examined the relationships among age, sex, length of hospitalization, presenting symptoms, co-morbidities, Evans index, Graeb score, Glasgow Coma Score, survival, and ventriculoperitoneal shunt complications through univariate and bivariate analyses. The Shapiro-Wilk test was used to evaluate data distribution. Differences between groups were analyzed using the chi-square test for categorical variables and the Mann-Whitney U test for non-parametric variables.

Results

The median age of the participants was 54 years, with a male predominance (57.7%). Motor dysfunction was the most frequently reported symptom at presentation (48.1%). Among the 30 patients who developed hydrocephalus after intraventricular hemorrhage, 70% had communicating hydrocephalus. There was a substantial correlation between mortality and hydrocephalus type (P =0.020).

Conclusion

Intraventricular bleeding is associated with an increased risk of communicating hydrocephalus, with an incidence rate of 3% per person-year.

## Introduction

Intraventricular hemorrhage (IVH) is a medical condition characterized by bleeding into the ventricular system [1,2]. It mostly occurs as an extension of an intracerebral hemorrhage in adults [3].Hydrocephalus (HCP), one of the main sequelae of IVH, is defined as ventricular enlargement due to increased cerebrospinal fluid volume and reduced flow [2,4,5]. HCP can be classified into two main categories: communicating and non-communicating [6,7], with the corresponding prevalence in patients with IVH of 49.3% and 67%, respectively [1,8].The IVH grading system may help determine the severity of bleeding and the risk of complications [9,10]. However, the relationship between IVH and the development of communicating HCP remains unclear. In this study, we aimed to estimate the incidence of communicating HCP following IVH and to identify the associated risk factors. 

## Materials and methods

This retrospective record review study was conducted at King Abdulaziz University Hospital in Jeddah, Saudi Arabia, and included patients with IVH. Data were retrieved from the hospital’s computerized database from 2010 to 2022. The study was approved by the Research Ethics Committee at King Abdulaziz University Faculty of Medicine (approval number: 295-23). 

Participant selection

The medical records of 113 patients were inspected, excluding those of preterm infants with germinal matrix IVH and those with incomplete hospital records. Data were extracted and categorized into demographic and clinical characteristics. Demographic characteristics included age, sex, and survival. Clinical characteristics included the length of hospitalization, HCP type (communicating or non-communicating), duration of HCP development post-IVH, chronic disease history, presenting complications, ventriculoperitoneal shunt (VP-shunt) insertion status, and associated complication rates.

The Evan index was used to measure ventricular enlargement, with values > 0.3 indicative of HCP. Communicating and non-communicating HCP were differentiated based on the presence of obstruction as determined by a CT scan [11,12]. IVH severity was assessed via computed tomography scans using the semiquantitative Graeb scale [9]. The Glasgow Coma Scale (GCS) score was used to determine the level of consciousness, with scores of 3-8, 9-12, and 13-15 points indicative of “severe”, “moderate”, and “mild” states, respectively [13]. The incidence of communicating HCP per person-year was measured by dividing the number of new cases by the total person-years at risk [14].

Data analysis

Microsoft Excel (Microsoft Corporation, Redmond, Washington, United States) was used for data entry, and statistical analyses were conducted using the IBM SPSS Statistics for Windows, Version 23.0 (Released 2015; IBM Corp., Armonk, New York, United States). Categorical variables are expressed as counts (%), and non-parametric variables are expressed as medians (25-75 percentiles). The Shapiro-Wilk test was used to confirm normal data distribution. Differences between groups were analyzed using the chi-square test for categorical variables and the Mann-Whitney test for non-parametric variables. Survival was determined using the Kaplan-Meier curves and log-rank test (Mantel-Cox). Statistical significance was set at p-values of < 0.05, with a confidence interval of 95%.

## Results

Among 113 patients, 52 met the eligibility criteria and were included in this study. The median age of the participants was 54 years, with male participants (57.7%) outnumbering females (42.3%). Most (53.8%) patients survived, with a median length of hospitalization (LOH) of 12.5 days. The main presenting complications were motor dysfunction (48.1%), increased intracranial pressure (40.4%), and decreased levels of consciousness (38.5%) (Table 1). Most (42.3%) GCS scores indicated “mild” states. Among 40 patients with co-morbidities, hypertension was the prevailing condition (67.3%), followed by diabetes mellitus (36.5%). 

**Table 1:** Baseline characteristics, GCS, duration of HCP development post-IVH, presenting complications, and co-morbidities of all participants. The data has been represented as n (%) and median (IQR) GCS: Glasgow Coma Scale; IVH: intraventricular hemorrhage; LOH: length of hospitalization; CNS: central nervous system; ICP: intracranial pressure; LOC: level of consciousness; HCP: hydrocephalus

Characteristics	Values
Age (years), median (IQR)	54 (41.25-63.00)
Gender, n (%)	
Male	30 (57.7%)
Female	22 (42.3%)
Mortality	
Alive, n (%)	28 (53.8%)
Died, n (%)	24 (46.2%)
LOH (days), median (IQR)	12.5 (4.00-23.50)
Duration of HCP post-IVH, median (IQR)	6.5 (1.00-56.00)
GCS, n (%)	
Mild (13-15)	22 (42.3%)
Moderate (9-12)	11 (21.2%)
Severe (3-8)	17 (32.7%)
Comorbidities, n (%)	
No	12 (23.1%)
Yes	40 (76.9%)
Hypertension	35 (67.3%)
Diabetes Mellitus	19 (36.5%)
CNS diseases	5 (9.6%)
Heart diseases	6 (11.5%)
Dyslipidemia	1 (1.9%)
Hypothyroidism	1 (1.9%)
Hematological disorders	2 (3.8%)
Presenting complications, n (%)	
Motor dysfunctions	25 (48.1%)
Increased ICP	21 (40.4%)
Decrease LOC	20 (38.5%)
Infection	5 (9.6%)
Urinary symptoms	6 (11.5%)
Psychological disorders	4 (7.7%)
Speech abnormalities	7 (13.5%)
Decreased activities	3 (5.8%)
Sensory dysfunctions	2 (3.8%)

HCP was found in 30 patients over a period of 12 years; among them, 21 patients developed communicating HCP, and nine developed non-communicating HCP (Table 2). The median Evans and Graeb scores were 0.34 and 11 points, respectively. A VP-shunt was performed in 15 patients with HCP; of these, four developed complications, mostly shunt blockage (14.3%) (Table 3). Patients with HCP had a significant relation pertaining to age, as well as urinary symptoms (p=0.044 and p=0.033, respectively); moreover, mortality (p=0.020), LOH (p=0.035), motor dysfunction (p=0.017), and GCS scores (p=0.044) were associated with the HCP type. Neither age (p=0.319) nor co-morbidities (p=0.329) were associated with the type of HCP. Furthermore, non-significant associations were documented among the duration of HCP development post IVH, VP shunt complications, Evan’s index, and Graeb score.

**Table 2:** Baseline characteristics, presenting complications, and comorbidities of participants according to the presence or absence of HCP and its type. The data has been represented as n (%) and median (IQR) ^†^ p-value is considered significant (p<0.05) LOH: length of hospitalization; CNS: central nervous system; ICP: intracranial pressure; LOC: level of consciousness; HCP: hydrocephalus

Characteristics	Presence of HCP		p-value	Types of HCP (n=30)		p-value
	No (n=22)	Yes (n= 30)		Communicating HCP (n=21)	Non-Communicating HCP (n=9)	
Age (years), median (IQR)	46.50 (30.0-57.0)	59.50 (49.0-66.0)	0.044	60 (51.5-66.0)	54 (23-72)	0.319
Gender, n (%)			0.111			0.236
Male	16 (72.7%)	14 (46.7%)		8 (38.1%)	6 (66.7%)	
Female	6 (27.3%)	16 (53.3%)		13 (61.9%)	3 (33.3%)	
Mortality			0.845			0.020^†^
Alive, n (%)	11 (50%)	17 (56.7%)		15 (71.4%)	2 (22.2%)	
Died, n (%)	11 (50%)	13 (43.3%)		6 (28.6%)	7 (77.8%)	
LOH (days), median (IQR)	13.50 (4.00-22.50)	11.50 (3.00-35.50)	0.650	9 (2.5-15.5)	16 (3-85)	0.035^†^
Comorbidities, n (%)			0.778			0.329
No	6 (27.3%)	6 (20%)		3 (14.3%)	3 (33.3%)	
Yes	16 (72.7%)	24 (80%)		18 (85.7%)	6 (66.7%)	
Hypertension	14 (87.5%)	21 (87.5%)	1.000	16 (88.9%)	5 (83.3%)	1.000
Diabetes mellitus	5 (31.3%)	14 (58.3%)	0.175	11 (61.1%)	3 (50%)	0.665
CNS diseases	1 (6.3%)	4 (16.7%)	0.631	2 (11.1%)	2 (33.3%)	0.251
Heart diseases	2 (12.5%)	4 (16.7%)	1.000	4 (22.2%)	-	0.539
Dyslipidemia	-	1 (4.2%)	1.000	1 (5.6%)	-	1.000
Hypothyroidism	-	1 (4.2%)	1.000	1 (5.6%)	-	1.000
Hematological disorders	1 (6.3%)	1 (4.2%)	1.000	1 (5.6%)	-	1.000
Presenting Complications, n (%)						
Motor dysfunctions	11 (50%)	14 (46.7%)	1.000	13 (61.9%)	1 (11.1%)	0.017^†^
Increased ICP	12 (54.5%)	9 (30%)	0.135	5 (23.8%)	4 (44.4%)	0.389
Decrease LOC	10 (45.5%)	10 (33.3%)	0.549	6 (28.6%)	4 (44.4%)	0.431
Infection	3 (13.6%)	2 (6.7%)	0.639	1 (4.8%)	1 (11.1%)	0.517
Urinary symptoms, n (%)	-	6 (20%)	0.033^†^	6 (28.6%)	-	0.141
Psychological disorders	1 (4.5%)	3 (10%)	0.629	3 (14.3%)	-	0.534
Speech abnormalities	4 (18.2%)	3 (10%)	P=0.438	3 (14.3%)	-	P=0.534
Decreased activities	1 (4.5%)	2 (6.7%)	P=1.000	1 (4.8%)	1 (11.1%)	P=0.517
Sensory dysfunctions	-	2 (6.7%)	P=0.502	2 (9.5%)	-	P=1.000

**Table 3:** Evan index, GCS, Location of IVH, Graeb score, VP shunt and its complications among HCP patients (N= 30) The data has been represented as n (%) and median (IQR) ^†^ statistically significant VP: ventriculoperitoneal shunt; GCS: Glasgow Coma Score; IVH: intraventricular hemorrhage; HCP: intraventricular hemorrhage

Characteristics	HCP patients (n=30)	Communicating (n= 21)	Non-communicating (n=9)	p-value
Evan index, median (IQR)	0.34 (0.30-0.37)	0.35 (0.32-0.39)	0.33 (0.29-0.34)	0.149
GCS, n (%)				0.044^†^
Mild (13-15)	14 (48.3%)	13 (61.9%)	1 (12.5%)	
Moderate (9-12)	4 (13.8%)	2 (9.5%)	2 (25%)	
Severe (3-8)	11 (37.9%)	6 (28.6%)	5 (62.5%)	
Graeb Score, median (IQR)	11 (5.5-15)	14 (6-15)	7 (3.75-10.75)	0.074
VP shunt insertion, n (%)				1.000
No	15 (50%)	10 (47.6%)	5 (55.6%)	
Yes	15 (50%)	11 (52.4%)	4 (44.4%)	
VP shunt complications, n (%)				1.000
No	11 (73.3%)	8 (72.7%)	3 (75%)	
Yes	4 (26.7%)	3 (27.3%)	1 (25%)	
Shunt blockage, n (%)	2 (14.3%)	2 (20%)	-	1.000
Overshunting, n (%)	1 (7.1%)	-	1 (25%)	0.286
Brain herniation, n (%)	1 (7.1%)	-	1 (25%)	0.286
Intraventricular pneumoencephalus, n (%)	1 (7.1%)	1 (10%)	-	1.000
Intracranial hematoma, n (%)	1 (7.1%)	-	1 (25%)	0.286

**Figure 1:**
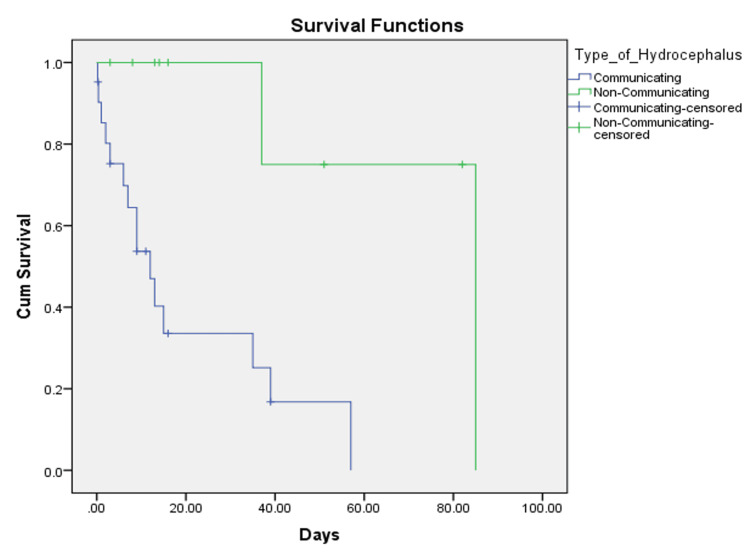
Kaplan-Meier curves displaying the estimated survival probability for patients with communicating and non-communicating HCP. Each vertical step in the curve indicates one or more events (i.e., deaths), and right-censored patients are indicated by a vertical mark in the curve at the censoring time. HCP: hydrocephalus

The log-rank test indicated that patients with non-communicating HCP had more favorable outcomes compared to patients with communicating HCP (p=0.007) (Figure 1). The incidence of communicating HCP was found to be 3% per person-year in adult patients with IVH. In summary, among 52 patients diagnosed with IVH, 30 had HCP. Of these, 70% had a communicating HCP.

## Discussion

IVH is a predictor of various neurological complications, with HCP being a primary concern. The etiologies of IVH, including factors such as advanced age, and co-morbidities such as hypertension and diabetes mellitus were examined to understand their impact on the incidence and development of HCP [15,16].

The present study aimed to ascertain the frequency of communicating HCP in patients who had experienced IVH. Our findings suggest a strong correlation between IVH and HCP development, with 57.7% of HCP cases presented in patients post IVH. Overall, communicating HCP was identified in 70% of the participants. Our observations align with those of Bu et al. [1] and Gluski et al. [17], who reported IVH-associated HCP incidence rates of 67% and 87%, respectively. We hypothesized that this elevated incidence was due to factors such as impaired cerebrospinal fluid resorption from blood breakdown products, inflammatory responses in the subarachnoid spaces, or altered cerebrospinal fluid dynamics due to ventricular bleeding [1,18].

 We did not observe a significant association between the duration of HCP development after IVH and the type of HCP. This indicates that the time it takes for HCP to develop post IVH does not differ significantly between communicating and non-communicating HCPs. We hypothesize that the lack of significant difference in timelines is due to the initial severity and extent of the hemorrhage, which disrupts cerebrospinal fluid absorption and flow in both types of HCP, resulting in similar onset times [19-21].

Our findings showed a significant correlation between post-IVH HCP and age (median, 54 years). Wang et al. [22] and Bhattathiri et al. [23] reported similar findings in patients with intracranial hemorrhage and a mean age of 62.34±10.85 and 62.9±12.2 years, respectively. In contrast, Alshardan et al., who focused on IVH specifically [24], and Diringer et al., who addressed all types of intracranial hemorrhage [8], reported contrasting findings. The increased risk in older adults may be explained by the fact that, as patients age, brain atrophy becomes more prevalent, resulting in an increased ventricular size and compliance. This structural change in the aging brain may impair cerebrospinal fluid dynamics, predisposing older adults to hydrocephalus following IVH. Additionally, the aging brain’s reduced capacity for repair, along with comorbid conditions such as small vessel disease, brain tumors, or infections, further increases the risk. Early intervention is crucial when such risks are identified to mitigate complications and improve outcomes [25-27].

We observed a significant association between LOH and mortality according to the HCP type. Furthermore, patients with non-communicating HCP had improved life expectancy compared to those with communicating HCP. Different studies have shown similar findings, concluding that communicating HCP is difficult to diagnose and treat [8,28]. Non-communicating HCP presents acutely and is addressed quickly, while communicating HCP is a chronic condition requiring long-term management and increases the risk of complications [29,30].

In this study, sex-based differences in the incidence of HCP were not observed, which is consistent with previous studies [8,22,24,31]. This uniformity may be attributed to similar anatomical structures and the absence of sex-specific hormones or factors influencing HCP incidence [32].

A noteworthy relationship was observed between the GCS score and HCP type in our study. We hypothesized that non-communicating HCP, due to obstruction in the cerebrospinal fluid pathway, might manifest with more immediate clinical deterioration compared to communicating HCP. This perspective is supported by several previous studies reporting similar findings [8,22,23,33]. Furthermore, our research indicated a substantial relationship between presenting complications such as urinary symptoms, motor dysfunction, and the presence and type of HCP. Excessive cerebrospinal fluid buildup in HCP can elevate intracranial pressure, leading to impaired motor function [34]. This link between urinary symptoms and motor dysfunction has been confirmed in other studies [35,36].

Contrary to our hypothesis, co-morbidities were not linked to communicating HCP following IVH, consistent with Alshardan et al. [24] regarding all co-morbidities and Diringer et al. [8] regarding diabetes mellitus. However, Diringer et al. found a significant association with hypertension, although the scope of these studies may have been broader, including different types of intracerebral hemorrhage [8]. This result suggests that the influence of co-morbidities might be more complex than initially anticipated and without a direct impact to guide the clinical approach.

Our study showed no significant association between Evans’ or Graeb’s scores and communicating HCP. Despite the initial hypotheses suggesting a correlation between a higher Evan index or Graeb score and an increased risk of communicating HCP, these scores might not be reliable predictors. Future research may integrate more factors or refine the scoring systems for a more comprehensive understanding.

Only 15 out of the 30 patients with HCP received VP shunts, while the remaining were managed with medical treatment and external ventricular drainage. The HCP type did not affect VP-shunt efficacy. The main VP shunt complication observed was shunt blockage. Additionally, we found one patient with non-communicating hydrocephalus who experienced multiple complications, including overshunting, brain herniation, and intracranial hematoma. These complications could be explained by the complex interplay of cerebrospinal fluid dynamics, shunt overdrainage, and subsequent intracranial pressure fluctuations. It is hypothesized that overshunting may have led to a rapid decrease in ventricular size, causing brain structures to shift and resulting in herniation and hematoma formation [37-39].

The limitations of our study include its single-center focus, small sample size, and reliance on potentially poor data sources, which may affect the generalizability and reliability of the findings. Additionally, the root cause of the higher incidence of communicating HCP compared to non-communicating HCP has not been thoroughly hypothesized or examined longitudinally or retrospectively. For a more comprehensive analysis of the incidence rate of HCP post IVH, we recommend conducting multicenter research with a larger, heterogeneous sample.

## Conclusions

Our study revealed a 3% incidence of communicating HCP per person-year in patients with IVH. Most affected patients did not survive, suggesting that the mortality risk associated with communicating HCP post IVH may be higher than that associated with non-communicating HCP. The latter's acute presentation might lead to a prompt intervention, whereas the former, due to its indolent nature, could manifest later, leading to further complications. We recommend regular post-discharge follow-up of patients.
